# QUARTIC: QUick pArallel algoRithms for high-Throughput sequencIng data proCessing

**DOI:** 10.12688/f1000research.22954.3

**Published:** 2020-10-08

**Authors:** Frédéric Jarlier, Nicolas Joly, Nicolas Fedy, Thomas Magalhaes, Leonor Sirotti, Paul Paganiban, Firmin Martin, Michael McManus, Philippe Hupé

**Affiliations:** 1 Institut Curie, Paris, F-75005, France; 2 U900, Inserm, Paris, F-75005, France; 3 PSL Research University, Paris, France; 4 Mines Paris Tech, Fontainebleau, F-77305, France; 5 Institut Pasteur, Paris, F-75015, France; 6 Université Paris Descartes, Paris, F-75006, France; 7 Intel Corporation, Hudson, Massachusetts, USA; 8 UMR144, CNRS, Paris, F-75005, France

**Keywords:** High-Throughput Sequencing, Alignment, Sorting, High-Performance Computing, MPI

## Abstract

Life science has entered the so-called 'big data era' where biologists, clinicians and bioinformaticians are overwhelmed with high-throughput sequencing data. While they offer new insights to decipher the genome structure they also raise major challenges to use them for daily clinical practice care and diagnosis purposes as they are bigger and bigger. Therefore, we implemented a software to reduce the time to delivery for the alignment and the sorting of high-throughput sequencing data.  Our solution is implemented using Message Passing Interface and is intended for high-performance computing architecture. The software scales linearly with respect to the size of the data and ensures a total reproducibility with the traditional tools. For example, a 300X whole genome can be aligned and sorted within less than 9 hours with 128 cores. The software offers significant speed-up using multi-cores and multi-nodes parallelization.

## Introduction

Since the first next generation sequencing technology was released in 2005 ( Kchouk *et al*., 2017), considerable progress has been made in terms of sequencing quality, diversity of protocols and throughput of the machines. As of today, the most recent generation of sequencers can easily produce terabytes of data each day and we expect this exponential growth of the sequencing to continue. This data tsunami raises many challenges, from data management to data analysis, requiring an efficient high-performance computing architecture ( Lightbody *et al*., 2019). Indeed, the throughput capacity of the sequencers tends to overwhelm the capacity of common computer architectures and the data analysis workflow to handle such amount of data in a reasonable time. As we have entered the era of genomic medicine, delivering the results to the clinicians within a short delay to guide the therapeutic decision is a challenge of the utmost importance in daily clinical practice. Several national initiatives worldwide such as France, USA, UK or Australia ( Stark *et al*., 2019) promote the use of genomics into healthcare. There is no doubt that exascale informatic architecture and software are required to tackle the challenges raised by the genomic medicine.

A typical bioinformatics workflow to analyze high-throughput sequencing (HTS) data consists of a set of systematic steps of pre-processing to i) align (or map) the sequencing reads on a reference genome and ii) to sort the alignments according to their coordinates on the genome. Those steps are fundamental for the efficiency and relevance of the downstream analysis to decipher genomic alterations such as mutations or structural variations. Traditional tools for aligning the reads are BWA-MEM ( Li & Durbin, 2010) and SOAP2 ( Li *et al*., 2009a), and the sorting is usually performed with Samtools ( Li *et al*., 2009b), Sambamba ( Tarasov *et al*., 2015), Picard tools and GATK ( McKenna *et al*., 2010). Most of the time, these steps are very time consuming (up to several days for whole genome analysis) as they suffer from bottlenecks at the CPU, IO and memory levels. Therefore, removing these bottlenecks would make it possible to reduce the time-to-delivery of the results such that they could be available within a reasonable delay when very large data are produced by the sequencers.

In order to tackle the aforementioned challenges to align and sort the sequencing data, we have developed software based on the Message Passing Interface (MPI) communication protocol ( Gropp *et al*., 1996) that makes it possible to fully benefit of parallel architecture of supercomputers ( Jarlier *et al*., 2020a; Jarlier *et al*., 2020b). MPI can therefore reduce the different bottlenecks by capitalizing on the low-latency network fabrics generally available on modern supercomputers. This allows an efficient distribution of the workload over the available resources of the supercomputers thus providing the expected scalability.

## Methods

### Alignment

**Figure 1.**
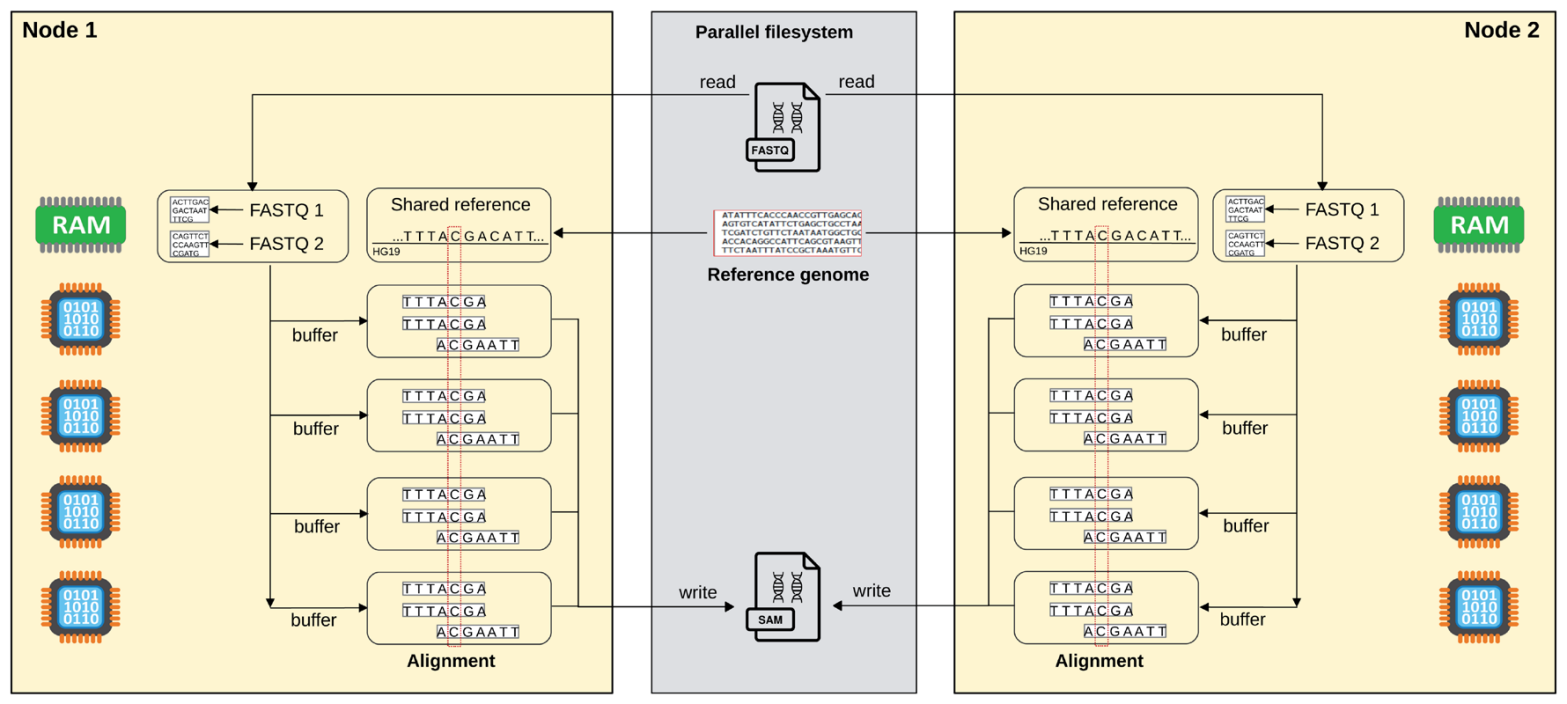
Alignment with mpiBWA using two computing nodes and four cores per node. Each core is in charge of aligning a chunk of the FASTQ file. The reference genome is stored in shared memory of each node of the computing cluster. Once aligned, the results are written into a SAM file. To ensure scalability, the read and write operations require a parallel filesystem.

**Figure 2.**
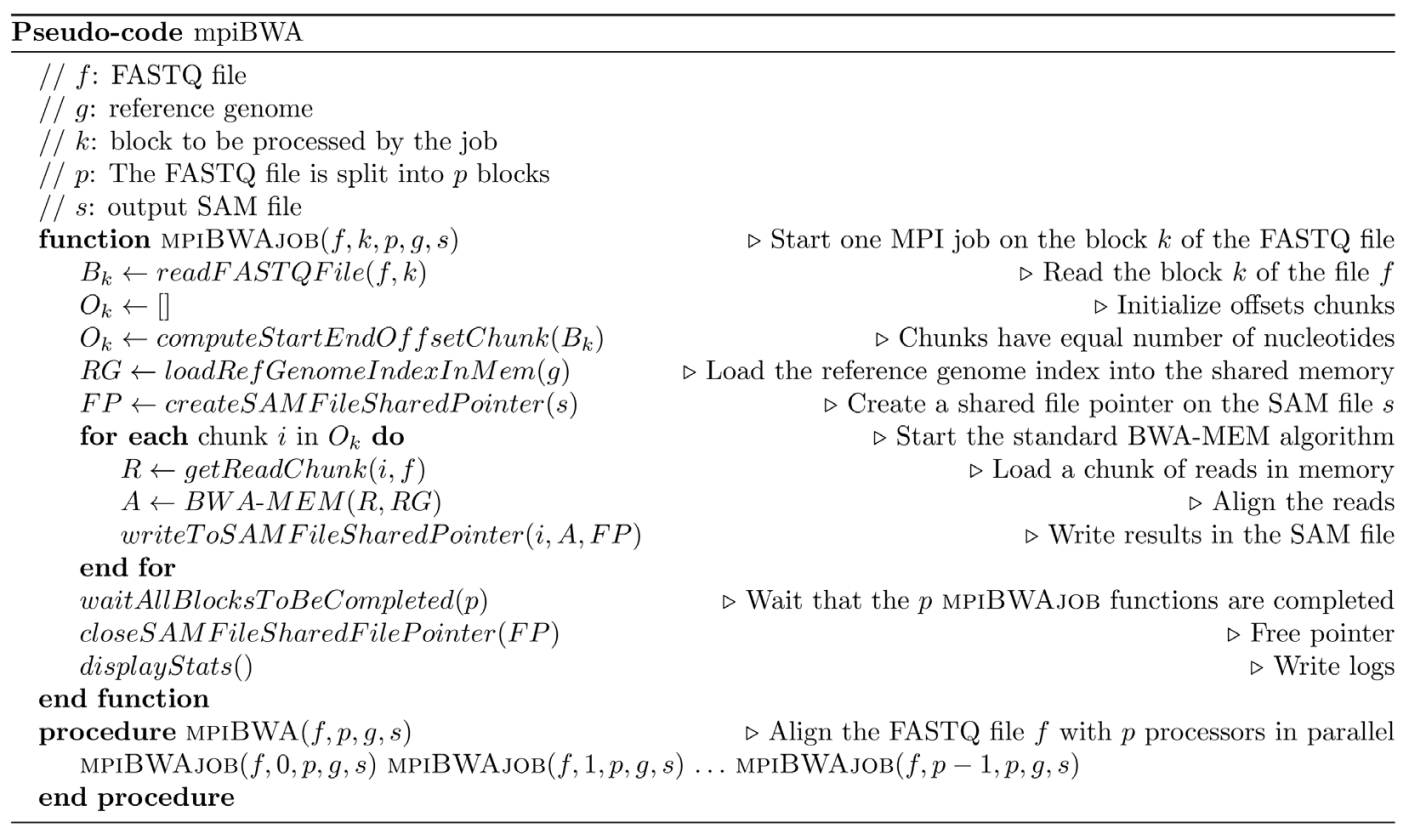
Pseudo-code of the mpiBWA which launches the function mpiBWAjob in parallel over p processors.

For the alignment, the software consists of a MPI wrapper to the BWA-MEM software such that the original code remains unchanged. The wrapper uses parallel IO and shared memory ( Figure 1). It first parses the input FASTQ files in order to define chunks of equal size. The different chunks are actually represented as two offsets (start and end) from the original FASTQ file thus reducing the amount of information to store. The reference genome of your choice is loaded into the shared memory on each computer node. Then, each chunk is processed in parallel threads by the original BWA-MEM algorithm. The results are finally written thanks to a shared file pointer in a unique SAM file. The pseudo-code is available in ( Figure 2). For a human genome, the size of the reference genome file in the shared memory takes around 5 GB and the total memory used by a BWA-MEM thread is around 300 MB. The original BWA-MEM processes a chunk of reads at a time that contains the same number of nucleotides which is also the case with our parallel implementation in order to allow the reproducibility with the original algorithm. We name our code alignment mpiBWA ( Jarlier *et al*., 2020a).

### Sorting

For the sorting, the software implements a parallel version of the bitonic sort proposed by Batcher (1968) for sorting any sequence of elements of size *n* = 2 *^k^* being of power of 2. Its complexity is ( *n* log ^2^ *n*) that is higher than ( *n* log *n*) from the popular merge sort algorithm. However, the bitonic sort is very suitable for parallel implementation since it always compares elements in predefined sorting network that is independent of the input data. To understand how the algorithm works, we first defined in what follows some concepts (see Grama *et al*. (2003) for details).

The algorithm relies on a *bitonic sequence* that is a sequence of values 〈 *a*_0_, *a*_1_, … , *a*_*n*-1_〉 with the property that 1) there is an index *i*, 0 ≤ *i* ≤ *n*-1 such that 〈 *a*_0_, *a*_1_, … , *a _i_*〉 is monotonically increasing and 〈 *a*_*i*+1_, … , *a*_*n*-1_〉 is monotonically decreasing, or 2) there exists a cyclic shift of indices so that the condition 1) is satisfied. From any bitonic sequence *s* = 〈 *a*_0_, *a*_1_, … , *a*_*n*-1_〉, the *bitonic split* operation consists in transforming the input sequence *s* into these two subsequences:

$$\begin{array}{l} s_{1}=\langle \text{min}(a_{0},a_{n/2}),\min(a_{1},a_{n/2+1}),...\;,\text{min}(a_{n/2-1},a_{n-1})\rangle \\ s_{2}=\langle \text{max}(a_{0},a_{n/2}),\max(a_{1},a_{n/2+1}),...\;,\text{max}(a_{n/2-1},a_{n-1})\rangle \end{array}$$

**Figure 3.**
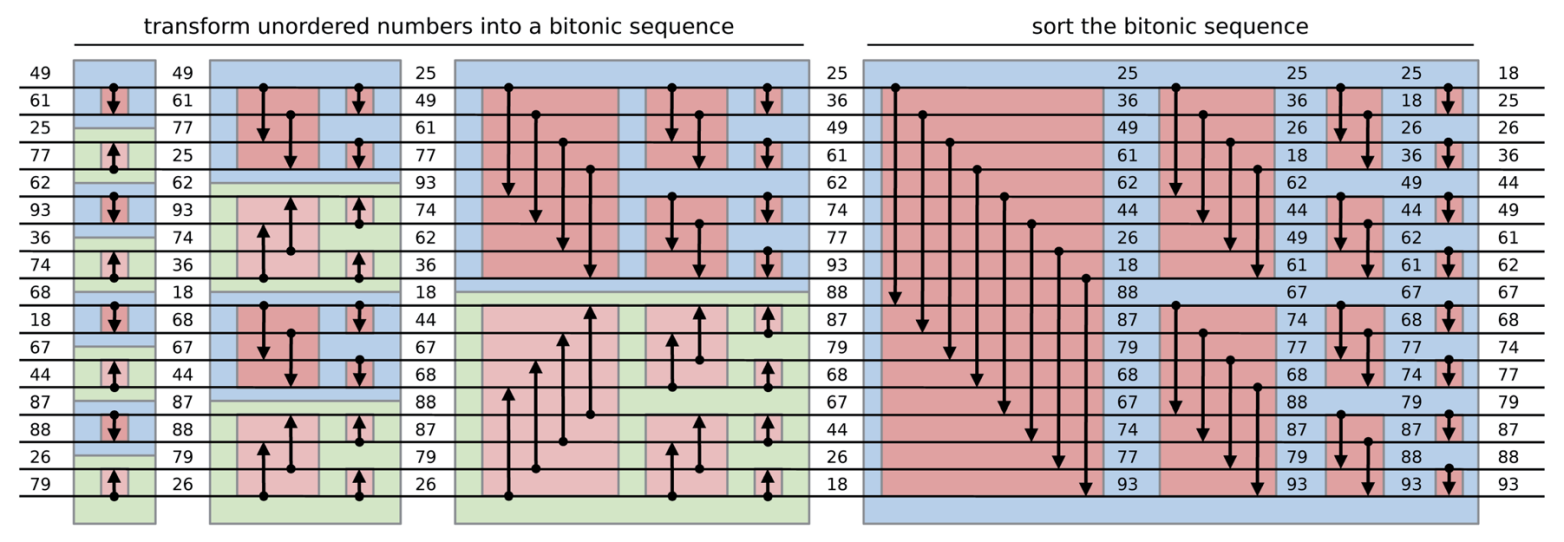
Sorting network for the bitonic sort. Each horizontal line represents an input and each arrow represents a two-input/two-output comparator which performs *compare-exchange* operation. Depending on its direction, the outputs are sorted in increasing (decreasing) order with a downwards (upwards) arrow. First, the comparator network transforms an input sequence of 16 unordered numbers into a bitonic sequence alternating $\text{B}{\text{M}}_{2}^{⊕}/\text{B}{\text{M}}_{2}^{⊖},$ $\text{B}{\text{M}}_{4}^{⊕}/\text{B}{\text{M}}_{4}^{⊖}\text{and}$ $\text{B}{\text{M}}_{8}^{⊕}/\text{B}{\text{M}}_{8}^{⊖}$ bitonic merges. Then, the obtained bitonic sequence is sorted with a recursive bitonic merge procedure $\text{B}{\text{M}}_{16}^{⊕}$ (adapted from Grama *et al*. (2003)).

Batcher (1968) proved that both *s*_1_ and *s*_2_ are bitonic sequences and the elements of *s*_1_ are smaller than the elements of *s*_2_. Thus, a recursive bitonic split from a bitonic sequence of size *n* = 2 *^k^*, until the sequence obtained are of size one allows the sorting of the input bitonic sequence in *k* splits as shown in Figure 3. The procedure of sorting a bitonic sequence from bitonic split operation is called *bitonic merge*. For each split of a bitonic merge, *n/*2 comparisons are performed during which the two numbers are exchanged if not in the right order using a *compare-exchange* operation. A bitonic merge procedure of a sequence of size *n* is noted $\text{B}{\text{M}}_{n}^{⊕}$ if the comparisons sort the number in monotonically increasing order or $\text{B}{\text{M}}_{n}^{⊖}$ for monotonically decreasing order.

Sorting any sequence of unordered number thus requires to convert the input sequence into a bitonic sequence. This is obtained using a *bitonic sorting network* (see Figure 3) that sorts *n* = 2 *^k^* numbers using *k -* 1 bitonic sorting stages, where the *i*-th stage is composed of *n/*2 *^i^* alternating increasing $\text{B}{\text{M}}_{2^{i}}^{⊕}$ and decreasing $\text{B}{\text{M}}_{2^{i}}^{⊖}$ bitonic merges. The final stage being $\text{B}{\text{M}}_{n}^{⊕}$ to obtain the sorted sequence.

**Figure 4.**
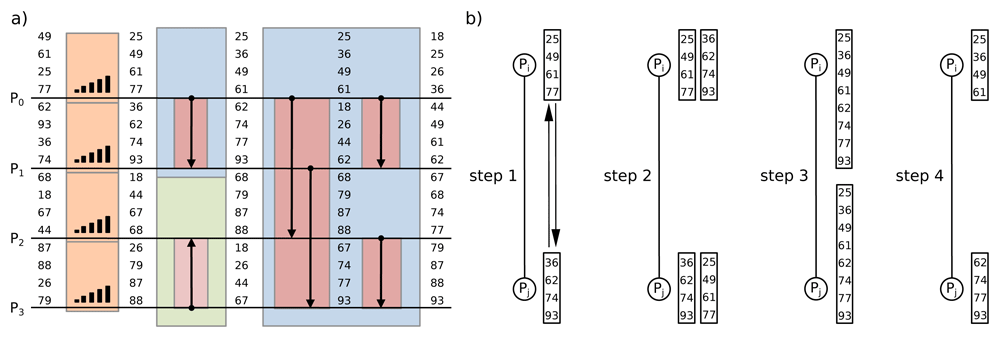
Parallel bitonic sort of 16 elements with four processors. ( **a**) Each horizontal line represents an input of elements attached to a processor. Each processor first locally sorts its list of 4 elements, then, the parallel sort performs three steps of *compare-split* operations over the bitonic sorting network. ( **b**) During the *compare-split* operation, each pair of processors first exchanges their information: each processor sends its block to the other process, then it merges the two sorted blocks into one list and stores only the appropriate half of the merged block to be used in the next step (adapted from Kim *et al*. (2001) & Grama *et al*. (2003)).

It is straightforward to generalize the bitonic sort algorithm on parallel architecture as shown in Figure 4. Each processor is in charge of an even number *m* = *n/p* elements (where *p* is the number of processors that is a power of 2) that are first sorted using a merge sort algorithm. Then, each comparison over the bitonic sorting network is replaced by a pair of processors which performs a *compare-split* operation. During the *compare-split* operation, the two sorted sequences from each processor are merged into one monotonic sorted list, then bisected into two (lower and higher) sequences. After the *compare-split*, one processor will keep the lower *m* elements from sequence and the other processor will keep the higher *m* elements according to the direction of the arrow in the step (see Kim *et al*. (2001) & Grama *et al*. (2003) for details). This parallelization allows the sorting of *n* elements in (log ^2^ *n*) time using a *bitonic sorting network*.

**Figure 5.**
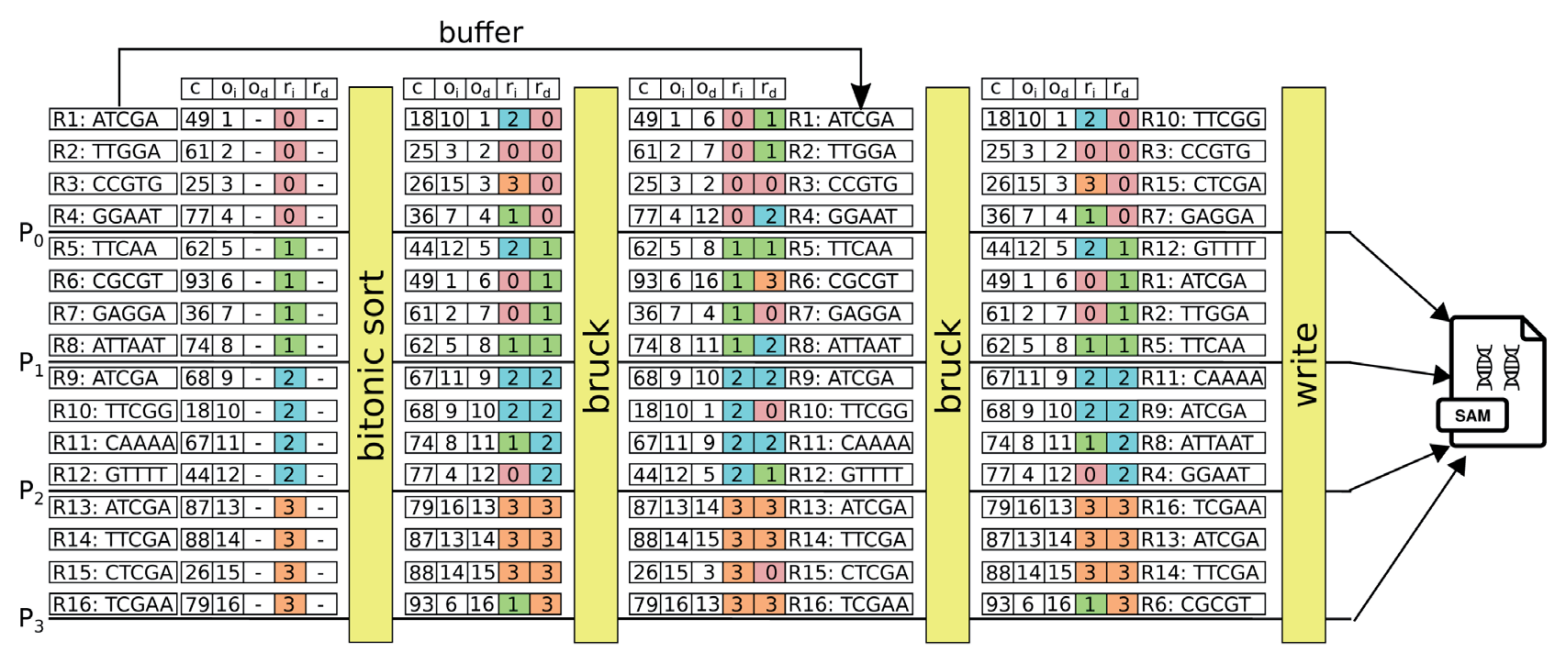
All-to-all communications with the Bruck algorithms for the sorting of 16 reads on four processors. The SAM file is read and split over the 4 processors P _0_ to P _3_ that sort the data with the bitonic sort. Then, a first Bruck phase sent the *r*_*d*_ and *o*_*d*_ values back to processor from where the data originated. The data from the SAM file are sent by a second Bruck phase to the processor that has been assigned to write the contiguous blocks (during this step, the original data is copied into the memory buffer and then exchanged). Finally, the data can be written on a parallel filesystem. *c*: genomic coordinate. *r _i_*: rank of the processor that parsed the block of the input SAM file. *o _i_*: offset of the line from the input SAM file. *r _d_*: rank of the processor that will write the block of the sorted data in the destination SAM file. *o _d_*: offset of the line in the sorted destination SAM file.

**Figure 6.**
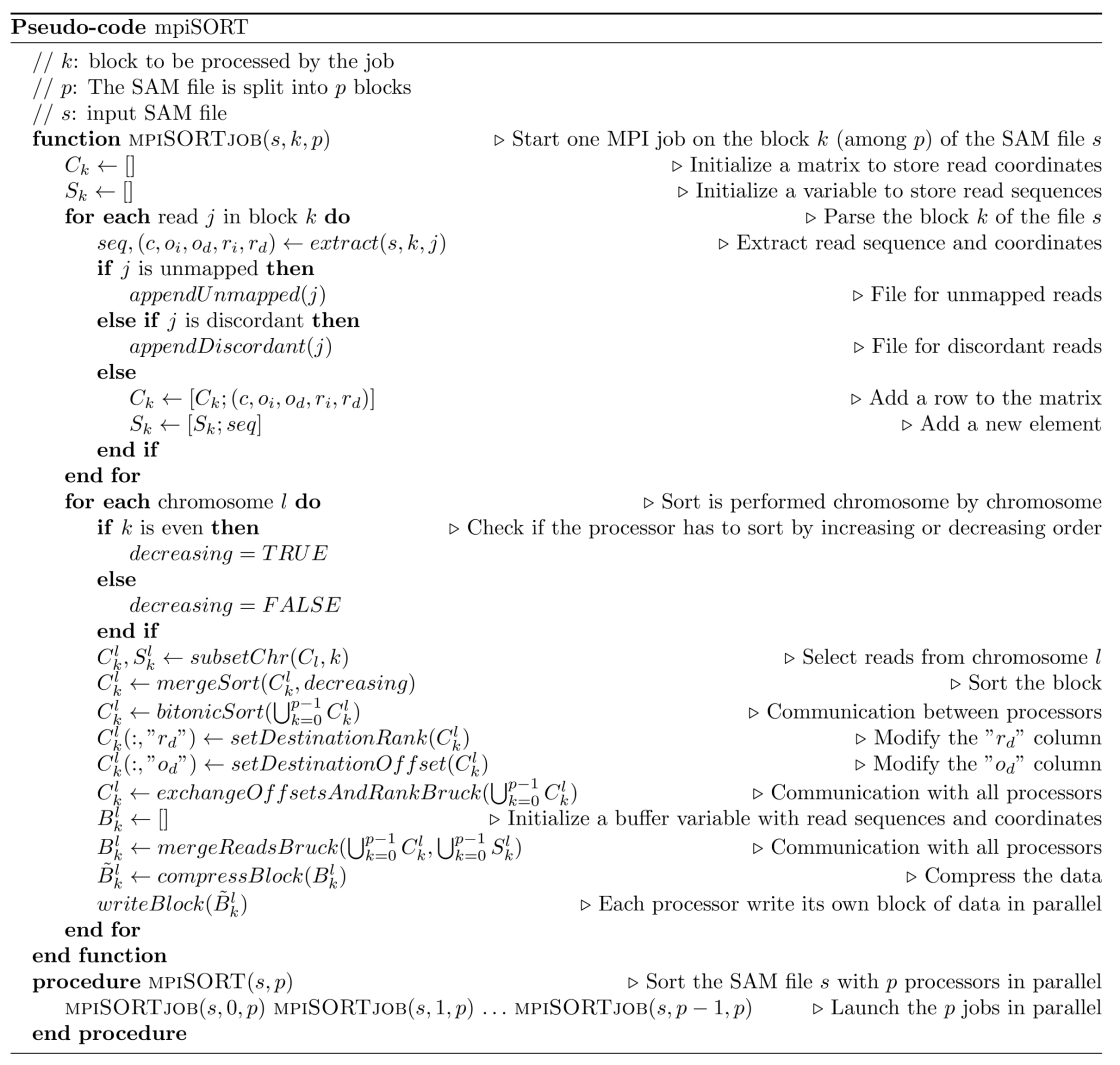
Pseudo-code of the mpiSORT which launches the function mpiSORTjob in parallel over p processors.

The workflow for the sorting is described in Figure 5 and the pseudo-code is available in Figure 6. The SAM file is read and split into *p* blocks that are dispatched across the *p* processors. Each block is parsed such that for each line of the SAM file, we extract the genomic coordinates of each read, its sequence (and all other information contained in the SAM file) and the line is indexed according to its offset in the SAM memory buffer of a processor. Then, the genomic coordinates are sorted with the bitonic sort as shown in Figure 4. During the sorting, a vector with five values follows the bitonic sorting network including the genomic coordinate *c*, the rank *r _i_* of the processor that parsed the block of the input SAM file, the offset *o _i_* of the line from the input SAM file, the rank *r _d_* of the processor that will write the block of the sorted data in the destination SAM file and the offset *o _d_* of the line in the sorted destination SAM file. Obviously, the values *r _d_* and *o _d_* are known when the bitonic sort is completed. It is important to highlight that the parallel computation is performed by different processors that are located on different compute nodes. This means that a block of data that has been read by a given processor is not accessible by another processor. Moreover, to optimize the writing of the sorted destination SAM file, it is essential to write the data in contiguous block. Thus, it is necessary that a processor in charge of the writing owns locally the data from a block of contiguous offsets *o _d_* in the sorted destination file. This implies that all the data have to be shuffled across all the processors that have to exchange data in an all-to-all communication step. To optimize the communication between the processors during the shuffle, we have implemented a Bruck algorithm ( Bruck *et al*., 1997) of time complexity (log ^2^ *p*). The Bruck algorithm is performed twice as shown in Figure 5 and can be seen as a joint procedure between two tables (the elements of the table being located on different processors). During the first Bruck phase, the *r _d_* and *o _d_* are sent back to the corresponding processor from where the data originated, and then, during the second Bruck phase, all the data (i.e. the appropriate lines of the SAM file) are sent to the processor that has been assigned to write the data. During the second phase the original data is copied into the memory buffer and then exchanged.

Note that when the SAM file contains several chromosomes, each chromosome is sorted successively. Therefore, the upper memory bound depends on the size of the whole SAM file plus internal structures plus the size of the biggest chromosome. In this case, the total amount of memory used is around 1.5 the size of the original SAM file if the file contains reads on all the chromosomes of the human genome. If the SAM file contains only one chromosome, then the total amount of memory required is 2.5 the size of the original SAM file. To be efficient, the sorting requires a number of cores being a power of 2. We name our code for sorting mpiSORT ( Jarlier *et al*., 2020b).

### Benchmark

We benchmarked mpiBWA and mpiSORT on the HG001 NA12878 sample from GIAB ( Zook *et al*., 2014). This sample is a whole genome with 300X depth of coverage composed of 2.13 billions 2x250 pair-ended reads. The BAM file has been downloaded from:

**url**: ftp://ftp-trace.ncbi.nlm.nih.gov/ReferenceSamples/giab/data/NA12878/

**folder**: NIST_NA12878_HG001_HiSeq_300x/NHGRI_Illumina300X_novoalign_bams/

The BAM file has been converted into FASTQ files using bedtools bamtofastq. In order to test the scalability of our software with respect to the size of the data, we downsampled the original 300X sample to obtain FASTQ files corresponding to depths of coverage ranging from 28X to 300X with a geometric progression with a common ratio of 1.6. The sequences have been aligned on the human genome GRCh38.

We ran the benchmark on a computing cluster equipped with Intel Xeon ^®^ Gold 6148 CPU @ 2.40GHz (Skylake architecture). The nodes are interconnected with Intel ^®^ Omni Path Architecture (OPA) of 100 Gbps speed. The parallel file system is BeegFS with two servers. We compile the programs with GCC 8.3 and use Open MPI 3.1.4. Jobs have been submitted using slurm scheduler.

### Implementation

The code has been written in C programming language using MPI directives. mpiBWA encapsulates the original BWA-MEM version 7.15. Two implementations for mpiBWA exist: the first outputs one SAM file with all the chromosomes, the second (named mpiBWAByChr) outputs one SAM file per chromosome. Moreover, mpiBWA comes along with mpiBWAIdx, which is responsible for creating a binary image of the reference genome. This image is subsequently loaded in the shared memory by mpiBWA. Then, every mpiBWA process on the same computing node will share the same genome reference in order to save memory usage. mpiBWAIdx does not need MPI to run.

### Operation

As our software rely on the MPI standard, mpirun must be available to run the program. Several MPI implementations exist such as mpich, open-mpi or Intel ^®^ MPI Library.

## Use cases

**Figure 7.**
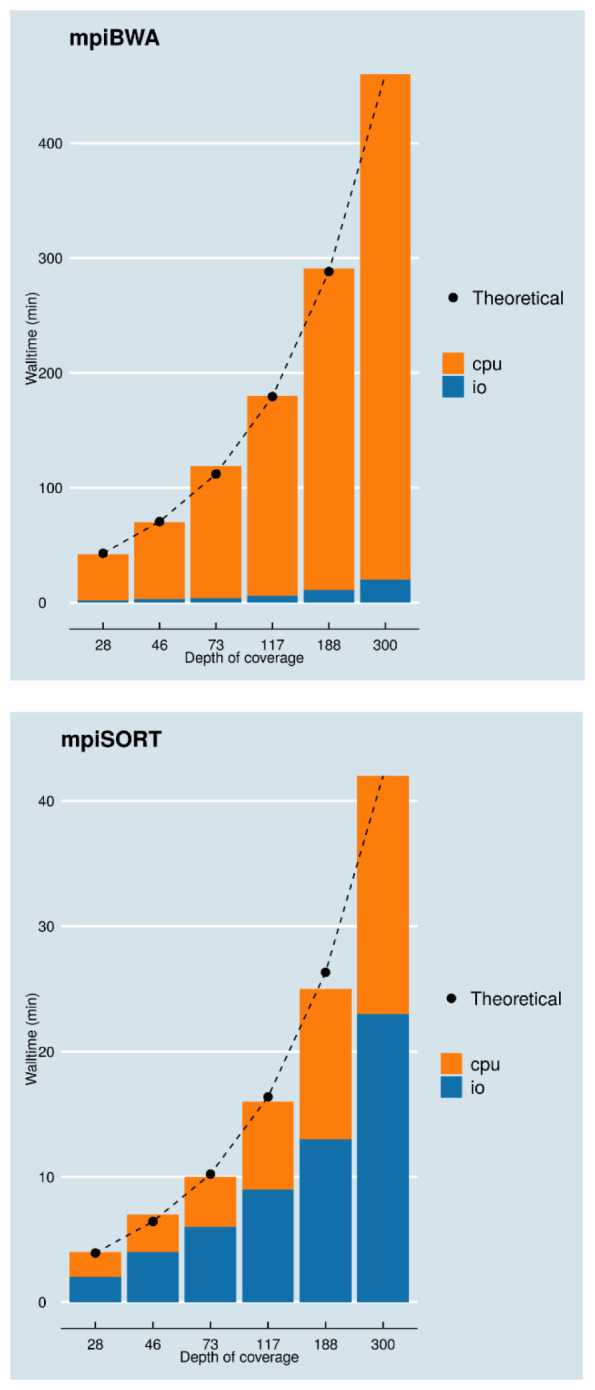
Walltimes for the alignment and sorting varying the sample sizes. The FASTQ files are first aligned with mpiBWAByChr, SAM files are generated as outputs and then sorted with mpiSORT and -u option. 128 cores have been used over 16 and four nodes with mpiBWA and mpiSORT, respectively. The black dots represent the theoretical values of the walltime with respect to the 300X depth of coverage being set as the reference.

The Figure 7 shows the scalability of both mpiBWA (with the mpiBWAByChr implementation) and mpiSORT with varying sample sizes (from 28X to 300X) using computation distributed over 128 cores. The output files from mpiBWAByChr have been used as input by mpiSORT. Both algorithms efficiently scale as the input data are bigger, the walltime to process the data being proportional to their input size. We assessed how much time was spent on pure computation versus IO ( *i.e.* reading the input files and writing the output files): mpiBWA spent more than 95% of its walltime in computation while mpiSORT spent between 50% to 60% in IO. The walltime to analyze the biggest sample of 300X is less than 8h hours for the alignment and less than one hour for the sorting.

**Figure 8.**
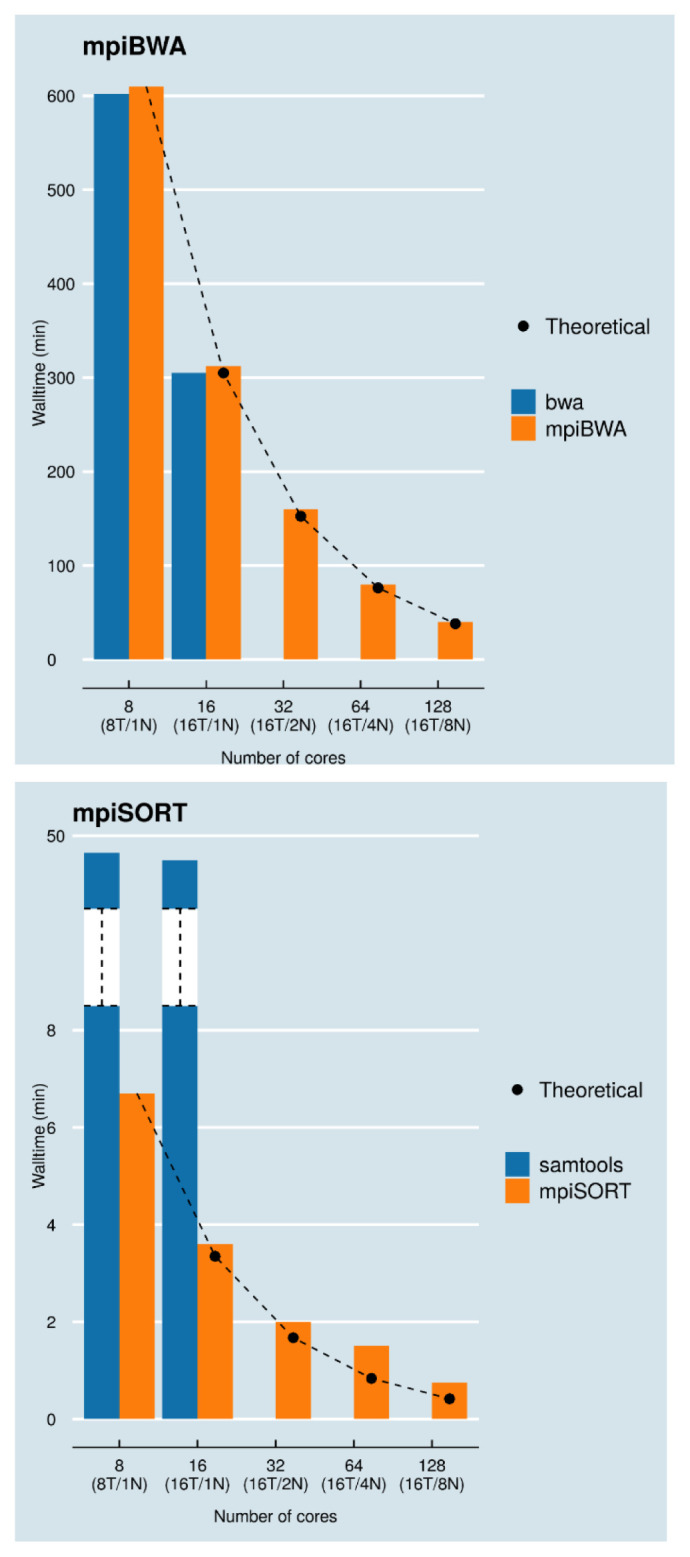
Walltimes for the alignment of the 28X depth of coverage sample and sorting of the chr16 from the 300X depth of coverage sample varying the number of cores. The number in brackets indicates how many threads (T) on how many nodes (N) have been launched, the number of cores used being the product of the two values. The black dots represent the theoretical values of the walltime with respect to the 8 cores value of the MPI implementation being set as the reference.

The Figure 8 compares the performance of both mpiBWA (with the mpiBWAByChr implementation) and mpiSORT with varying number of cores and nodes with respect to the classical tools BWA-MEM version 7.15 and samtools version 1.10. On a single node, the walltimes are very similar between BWA-MEM and mpiBWA whatever 8 or 16 threads are used with BWA-MEM to process the 28X sample. With mpiBWA, increasing the number of cores, either on the same node or over multiple nodes (from 2 to 8) allowing the distribution of the computation up to 128 cores, shows a linear scalability (the walltime is divided by 2 when the number of cores is doubled) that follows the expected theoretical walltimes. For the sorting, mpiSORT is very efficient since it offers a speed-up of 6.4 over samtools using 8 threads (or cores) on a single node to process the SAM file of the chr16 from the 300X sample. Doubling the number of threads did not change the walltime with samtools. With mpiSORT, increasing the number of cores, either on the same node or over multiple nodes, shows a linear scalability.

### mpiBWA

The first step consists in building the binary image of the reference genome that will be used in the shared memory by mpiBWA:


mpiBWAIdx hg19.small.fa


This creates the file myReferenceGenome.fa.map.

Importantly, mpiBWAIdx requires the following files that are generated by BWA index:


  •  myReferenceGenome.fa.sa
  •  myReferenceGenome.fa.bwt
  •  myReferenceGenome.fa.ann
  •  myReferenceGenome.fa.pac
  •  myReferenceGenome.fa.amb


The .map file only needs to be generated once. Then, mpiBWA is executed with the mpirun program, for example:


mpirun -n 
                        2 mpiBWA mem -t 
                        8 -o ./HCC1187C.sam hg19.small.fa 
                        \
    HCC1187C_R1_10K.fastq 
                        \
    HCC1187C_R2_10K.fastq



This command launches two MPI processes with eight BWA threads each (16 cores will be used). If you want to split the results of the alignment by chromosome, use mpiBWAByChr, for example:


mpirun -n 
                        2 mpiBWAByChr mem -t 
                        8 -o ./HCC1187C.sam hg19.small.fa 
                        \
    HCC1187C_R1_10K.fastq 
                        \
    HCC1187C_R2_10K.fastq


The total memory used during the alignment if approximately the size of the .map file plus the size of the SAM chunks loaded by each BWA tasks. A BWA thread takes around 300 MB of memory.

### mpiSORT

From the results obtained with mpiBWAByChr, each SAM files can be sorted as follows:


mpirun -n 
                        4 mpiSORT -u chr1.sam 
                        ${HOME}/mpiSORTExample


This command launches four MPI processes.

As mpiSORT requires a the entire input SAM file to be loaded into the memory, the program is memory bounded. Therefore, a lot of attention has to be paid to define how many cores are needed to process the data. For example, let’s assume that the computing cluster consists of nodes with 190 GB of RAM memory with 40 cores each (thus 4.75 GB per core is available but this value can be rounded to the lower limit of 4.5 GB to leave some free memory on the node). To choose the number of cores, the following rule has to be applied: the total memory for sorting a SAM file that contains only one chromosome is around 2.5 times the size of the SAM file. For instance, sorting a chr1.sam file of size 209 GB with 4.5 GB per core requires 128 cores, for a of size 110 GB it requires 64 cores. We remind that the bitonic sort algorithm requires a number of cores that is a power of 2, this is the reason why the number of cores has to be set to the upper bound to the closest power of 2.

## Conclusion

In this paper, we described parallel algorithms ( Jarlier *et al*., 2020a; Jarlier *et al*., 2020b) to process sequencing data that fully benefit from high-performance architecture with a total reproducibility and linear speed-ups. Our implementation is based on bitonic sorting network, Bruck algorithm and IO optimizations with MPI directives. MPI efficiently removes POSIX barriers like the one-file per process or thread concurrent access. Indeed, this differs from other implementations using embarrassingly parallel approaches ( Decap *et al*., 2015; Kawalia *et al*., 2015; Puckelwartz *et al*., 2014) that rely on the MapReduce paradigm: in this case, input files are first split into small chunks, each one being analyzed by a process, and the different output files are then merged into a single output. With MPI, splitting the input file is not necessary since it can efficiently handle concurrent access on a single file.

The scalability of the software strongly relies on the underlying hardware architecture, such as the low latency interconnection and parallel filesystem. This implies that the hospitals or institutions need a powerful state-of-the-art informatic architecture that is either available locally or provided with mutualized resources through national infrastructures that are certified to process healthcare data. Interestingly, our implementation allows the generation of aligned read files for each chromosome that can be further sorted in parallel thus reducing the time of downstream analysis whenever an analysis per chromosome is possible. The performance we obtained showed that MPI is very relevant for the field of genomics. The tools we developed pave the way towards the use of whole genome sequencing in daily clinics in order to meet the deadline and deliver results to the clinician in real-time for precision medicine as the time to delivery can be reduced to several minutes if the processing is distributed over several hundreds of cores.

## Data availability

All data underlying the results are available as part of the article and no additional source data are required.

## Software availability

**Source code for mpiBWA available at**: https://github.com/bioinfo-pf-curie/mpiBWA.

**Archived source code at time of publication**: https://doi.org/10.5281/zenodo.3887185 ( Jarlier *et al*. (2020a)).

**Source code for mpiSORT available at**: https://github.com/bioinfo-pf-curie/mpiSORT.

**Archived source code at time of publication**: http://www.doi.org/10.5281/zenodo.4061917 ( Jarlier *et al*. (2020b)).

**All documentation is available at**: https://github.com/bioinfo-pf-curie/QUARTIC.

**License**: CeCILL Version 2.1.
