# Supplementary material for: QUARTIC: QUick pArallel algoRithms for high-Throughput sequencIng data proCessing
Source: F1000Res. 2020 Oct 8;9:240. Originally published 2020 Apr 6. [Version 3] doi: 10.12688/f1000research.22954.3 (PMC7429925; doi:10.12688/f1000research.22954.3)
Supplement: Peer review history

# Peer review: Reviewer response for version 3

**DOI:** 10.5256/f1000research.30033.r72713

**Published:** 2020-10-12

**Authors:** Salvador Capella-Gutierrez, Ramon Amela Milian

**Affiliations:** 1 Computational Genomics. Life Sciences Department, Barcelona Supercomputing Center (BSC), Barcelona, Spain; 2 Spanish National Bioinformatics (INB) Coordination Node, Barcelona Supercomputing Center, Barcelona, Spain

We don't have further comments.

Are the conclusions about the tool and its performance adequately supported by the findings presented in the article?

Partly

Is the rationale for developing the new software tool clearly explained?

Yes

Is the description of the software tool technically sound?

Yes

Are sufficient details of the code, methods and analysis (if applicable) provided to allow replication of the software development and its use by others?

Yes

Is sufficient information provided to allow interpretation of the expected output datasets and any results generated using the tool?

Partly

Reviewer Expertise:

bioinformatics

We confirm that we have read this submission and believe that we have an appropriate level of expertise to confirm that it is of an acceptable scientific standard.

# Peer review: Reviewer response for version 3

**DOI:** 10.5256/f1000research.30033.r72714

**Published:** 2020-10-09

**Authors:** Yupu Liang

**Affiliations:** 1 Research Bioinformatics, CCTS, The Rockefeller University, New York, NY, USA

I am satisfied with the response and have no further comments.

Are the conclusions about the tool and its performance adequately supported by the findings presented in the article?

Yes

Is the rationale for developing the new software tool clearly explained?

Yes

Is the description of the software tool technically sound?

Yes

Are sufficient details of the code, methods and analysis (if applicable) provided to allow replication of the software development and its use by others?

Partly

Is sufficient information provided to allow interpretation of the expected output datasets and any results generated using the tool?

Yes

Reviewer Expertise:

bioinformatics and medical informatics

I confirm that I have read this submission and believe that I have an appropriate level of expertise to confirm that it is of an acceptable scientific standard.

# Peer review: Reviewer response for version 2

**DOI:** 10.5256/f1000research.27364.r67518

**Published:** 2020-08-13

**Authors:** Yupu Liang

**Affiliations:** 1 Research Bioinformatics, CCTS, The Rockefeller University, New York, NY, USA

Jarlier and co-authors developed an efficient method of short reads mapping and sorting through utilizing Message Passing Interface(MPI) . Specifically, they implement an MPI wrapper to the well-known mapper BWA-MEM existing method, named mpiBWA. They then implement a parallel version of the sorting method called mpiSORT through the implementation of bitonic sort. The authors have shown mathematically that their sorting method is �� (log2 *n*) time. They have also done benchmark experiments to compare their software to BWA and samtools. They demonstrated that their tools could archive linear scalability by adding computing cores to the system. The authors did a great job of explaining the process through figures. They have also provided sample command lines to use the software in action. I do wish the authors would could also provide pseudo-code of their implementation, which will help readers to understand the process better. Another minor point is that given the software's performance strongly relies on the underlying hardware architecture, and it would also be helpful for the authors to provide helper script(s) that help to calculate the number of cores to use on particular data input and computing infrastructure.

 In conclusion, the authors demonstrate that using bitonic sorting network, Bruck algorithm and IO optimizations with MPI directives can improve the performance of sequencing data handling.

Are the conclusions about the tool and its performance adequately supported by the findings presented in the article?

Yes

Is the rationale for developing the new software tool clearly explained?

Yes

Is the description of the software tool technically sound?

Yes

Are sufficient details of the code, methods and analysis (if applicable) provided to allow replication of the software development and its use by others?

Partly

Is sufficient information provided to allow interpretation of the expected output datasets and any results generated using the tool?

Yes

Reviewer Expertise:

bioinformatics and medical informatics

I confirm that I have read this submission and believe that I have an appropriate level of expertise to confirm that it is of an acceptable scientific standard.

# Response

**Published:** 2020-10-02

**Authors:** Philippe Hupé

**Affiliations:** Institut Curie, France

First, we would like to thank the second reviewer. We are very grateful for

 her time and very valuable comments that significantly helped to

 improve the article and the documentation of the tools.

 You will find below a detailed answer to the different points that we have

 addressed in the revised manuscript.

 Best regards,

 Frédéric Jarlier and Philippe Hupé

**Detailed response to the reviewer2's comments**

 > I do wish the authors would could also provide pseudo-code of their implementation, which will help readers to understand the process better.

 Indeed to facilitate the reading and understanding of both mpiBWA and mpiSORT

 algorithms, we added 2 figures with the pseudo-code.

 > Another minor point is that given the software's performance strongly relies on the underlying hardware architecture, and it would also be helpful for the authors to provide helper script(s) that help to calculate the number of cores to use on particular data input and computing infrastructure

 In order to help the user when writing submission scripts to a computing

 cluster, we added two scripts in the example folder of the github repository

 of mpiSORT.

 These scripts allow the users to explore the different partitions of the

 computing cluster (getSlurmNodesInfo.sh) and give advices in term of resources needed to run the MPI program (informaticResources.py).

 We also updated the benchmarking section of the documentation with examples.

# Peer review: Reviewer response for version 2

**DOI:** 10.5256/f1000research.27364.r65398

**Published:** 2020-07-14

**Authors:** Salvador Capella-Gutierrez, Ramon Amela Milian

**Affiliations:** 1 Computational Genomics. Life Sciences Department, Barcelona Supercomputing Center (BSC), Barcelona, Spain; 2 Spanish National Bioinformatics (INB) Coordination Node, Barcelona Supercomputing Center, Barcelona, Spain

Thanks to the authors for addressing point-by-point each comment. We have no further comments to make.

Are the conclusions about the tool and its performance adequately supported by the findings presented in the article?

Partly

Is the rationale for developing the new software tool clearly explained?

Yes

Is the description of the software tool technically sound?

Yes

Are sufficient details of the code, methods and analysis (if applicable) provided to allow replication of the software development and its use by others?

Yes

Is sufficient information provided to allow interpretation of the expected output datasets and any results generated using the tool?

Partly

Reviewer Expertise:

bioinformatics

We confirm that we have read this submission and believe that we have an appropriate level of expertise to confirm that it is of an acceptable scientific standard.

# Peer review: Reviewer response for version 1

**DOI:** 10.5256/f1000research.25341.r62078

**Published:** 2020-05-04

**Authors:** Salvador Capella-Gutierrez, Ramon Amela Milian

**Affiliations:** 1 Computational Genomics. Life Sciences Department, Barcelona Supercomputing Center (BSC), Barcelona, Spain; 2 Spanish National Bioinformatics (INB) Coordination Node, Barcelona Supercomputing Center, Barcelona, Spain

Jarlier and colleagues introduce their work on parallelizing popular bioinformatics programs, which have become the bottleneck for the current scenario of massive NGS data generation even in clinical settings.

 Authors describe two MPI implementations for the alignment and sorting steps used as part of the NGS data preprocessing in order to better take advantage of parallel architectures. Algorithms are well described and the documentation is good, making really easy the reproducibility of their results ^1^. In addition, authors provide software containers (singularity), which can be used in controlled environments including HPC installations.

 Despite the existing benchmarking efforts carried by authors, a scalability study is missing. Ideally, benchmarking should include varying resources allocation so readers can benefit from such comparison. We would like to suggest, as you have done with varying deep coverage sizes, you make the scalability study through a varying number of processors e.g. 4, 8, 16, 32, 64, 128. It would be also interesting to use as a baseline the normal execution of those programs to illustrate the improvement of the MPI implementation regarding the standard approaches. This way, it would be possible to answer the following questions:

- Is the MPI version faster when using a single node?
- Otherwise, from which point is better to use the MPI version than the most common software. I.e. in case the MPI is 2 times slower but scales linearly, the execution time would be shorter when giving the MPI implementation at least 2 nodes.

This way, the potential users would know better the scenarios in which using this implementation becomes more interesting.

 You have mentioned there are similar approximations to this problem. However, you just mention them in the conclusions. Wouldn’t it make sense to discuss similarities and differences with other implementations in the text?

 Apart from the benchmarking component, it would be interesting to further discuss the scenarios where implementations like this one will be used. It is not clear that clinical settings will have internally very powerful computational facilities for their daily use.

 Finally, we would like to mention some minor points related to the text itself.

**Abstract:** Moving from the general problem to the specific solution without mentioning that the NGS data preprocessing (alignment and sorting) represent major bottlenecks to timely results deliver for diagnostic use.

**Methods:**

- **Alignment**.  reference genome file > indicate a version.
- **Sorting section**. Apparently there is a miswriting of the theoretical computational cost of the algorithm > O (n log2 n) that is higher than O (n log n).

**Minor:**

- **Introduction**: data each day s > data each day.
- **Introduction**:  align and sort the sequencing data, we developed software >  align and sort sequencing data, we **have** developed software.
- **Sorting section**: by a pair of processors with performs a compare-split operation > by a pair of processors **which** performs a compare-split operation.

Are the conclusions about the tool and its performance adequately supported by the findings presented in the article?

Partly

Is the rationale for developing the new software tool clearly explained?

Yes

Is the description of the software tool technically sound?

Yes

Are sufficient details of the code, methods and analysis (if applicable) provided to allow replication of the software development and its use by others?

Yes

Is sufficient information provided to allow interpretation of the expected output datasets and any results generated using the tool?

Partly

Reviewer Expertise:

bioinformatics

We confirm that we have read this submission and believe that we have an appropriate level of expertise to confirm that it is of an acceptable scientific standard, however we have significant reservations, as outlined above.

## References

[1] Reproducibility of Results.2020; Reference source

# Response

**Published:** 2020-06-12

**Authors:** Philippe Hupé

**Affiliations:** Institut Curie, France

First, we would like to thank the reviewers. We are very grateful for their time, contribution and very valuable comments that significantly helped to improve the article and the documentations of the tools.

 You will find below a detailed answer to the different issues that we have addressed in the revised manuscript.

 Best regards,

 Frédéric Jarlier and Philippe Hupé

**Detailed response to the reviewer1's comments**

 >  Despite the existing benchmarking efforts carried by authors, a scalability study is missing. Ideally, benchmarking should include varying resources allocation so readers can benefit from such comparison. We would like to suggest, as you have done with varying deep coverage sizes, you make the scalability study through a varying number of processors e.g. 4, 8, 16, 32, 64, 128.

 Indeed, this benchmark varying the number of cores was clearly missing. Therefore, a scalability study has been added for both mpiBWA and mpiSORT using 8, 16, 32, 64, 128 cores. The results of this benchmark are described in the section "Use cases" and in Figure 6. We took this opportunity of this new benchmark to use the last version (1.1) of our MPI implementations (Figure 5have been therefore updated).

 > It would be also interesting to use as a baseline the normal execution of those programs to illustrate the improvement of the MPI implementation regarding the standard approaches.

 As suggested, we also compared our MPI implementations with respect to a reference baseline using the traditional tools (bwa and samtools). The walltimes of the reference baseline are presented in the section "Use cases" and in Figure 6.

 > This way, it would be possible to answer the following questions:

 >  

 >      Is the MPI version faster when using a single node?

 >       

 The results show that the walltimes are similar between mpiBWA and bwa. mpiSORT is much faster than samtools offering a speed-up over 6.

 >      Otherwise, from which point is better to use the MPI version than the most common software. I.e. in case the MPI is 2 times slower but scales linearly, the execution time would be shorter when giving the MPI implementation at least 2 nodes.

 >  

 The results of the scalability benchmark and the comparison with respect to the traditional tools demonstrate that both mpiBWA and mpiSORT perform efficiently on a single node and on multiple nodes.

 >  This way, the potential users would know better the scenarios in which using this implementation becomes more interesting.

 >  

 The results of the scalability benchmark show that the MPI implementation can be very versatile.

 Our MPI implementations can easily address the two typical scenarii:

- when time-to-delivery matters, the scalability allows the processing of the data very quickly using multiple cores and nodes.
- when the throughput matters (i.e. number of samples that can be analyzed at the same time), you can process a sample on a single node.

We also added a benchmark section in the documentation of the github repository such that the user can reproduce the benchmark to figure out what is the best scenario according to the computing infrastructure that is used. The documentations on the github repository describe how to assess the memory and cpu usage for the traditional tools and the MPI implementations as we did for the scalabitity study. The documentation is available here:

- https://github.com/bioinfo-pf-curie/mpiSORT/blob/master/docs/README.md#benchmark
- https://github.com/bioinfo-pf-curie/mpiBWA/blob/master/docs/README.md#benchmark

>  You have mentioned there are similar approximations to this problem. However, you just mention them in the conclusions. Wouldn’t it make sense to discuss similarities and differences with other implementations in the text?

 We added in the conclusion that:

*Indeed, this differs from other implementations using embarrassingly parallel approaches (Puckelwartz et al., 2014; Decap et al., 2015; Kawalia et al.,2015} that rely on the MapReduce paradigm: in this case, input files are first split into small chunks, each one being analyzed by a process, and the different output files are then merged into a single output. With MPI, splitting the input file is not necessary.*

 >  Apart from the benchmarking component, it would be interesting to further discuss the scenarios where implementations like this one will be used. It is not clear that clinical settings will have internally very powerful computational facilities for their daily use.

 >

  We agree that the access to a powerful and up-to-date computing infrastructure may be a bottleneck. We added in the conclusion of manuscript:

*This implies that the hospitals or institutions need a powerful state-of-the-art informatic*

*architecture that is either available locally or provided  with mutualized resources through national infrastructures that are certified to process healthcare data.*

 >  Finally, we would like to mention some minor points related to the text itself.

 >  

 >  Abstract: Moving from the general problem to the specific solution without mentioning that the NGS data preprocessing (alignment and sorting) represent major bottlenecks to timely results deliver for diagnostic use.

 We agree that the Abstract needed to be improved. It has been substantially modified in the revised version.

 >  Methods:

 >  

 >      Alignment.  reference genome file > indicate a version.

 >      

 We added in the text " *The reference genome of your choice*" as the user can use any genome.

 >      Sorting section. Apparently there is a miswriting of the theoretical computational cost of the algorithm > O (n log2 n) that is higher than O (n log n).

 The complexity are correct. Its is O (n log2 n) for the bitonic sort, it is higher that other sort algorithms but it can be easily parallelized.

 >  

 >  Minor:

 >  

 >      Introduction: data each days > data each day.

 >       

 >      Introduction:  align and sort the sequencing data, we developed software >  align and sort sequencing data, we have developed software.

 >       

 >      Sorting section: by a pair of processors with performs a compare-split operation > by a pair of processors which performs a compare-split operation.

 >

 The typos have been corrected.
